# Diagnostic delay in axial spondyloarthritis: a systematic review

**DOI:** 10.1007/s10067-022-06100-7

**Published:** 2022-02-19

**Authors:** Charles A. Hay, Jon Packham, Sarah Ryan, Christian D. Mallen, Alexandros Chatzixenitidis, James A. Prior

**Affiliations:** 1 School of Medicine, Keele University, Keele, ST5 5BG UK; 2 Academic Unit of Population and Lifespan Sciences, University of Nottingham, Nottingham, UK; 3 Midlands Partnership NHS Foundation Trust, Stafford, UK; 4 School of Nursing and Midwifery, Keele University, Keele, ST5 5BG UK

**Keywords:** Ankylosing spondylitis, Axial spondyloarthritis, Diagnostic delay

## Abstract

Identification of axial spondyloarthritis (axSpA) remains challenging, frequently resulting in a diagnostic delay for patients. Current benchmarks of delay are usually reported as mean data, which are typically skewed and therefore may be overestimating delay. Our aim was to determine the extent of median delay patients’ experience in receiving a diagnosis of axSpA and examine whether specific factors are associated with the presence of such delay. We conducted a systematic review across five literature databases (from inception to November 2021), with studies reporting the average time period of diagnostic delay in patients with axSpA being included. Any additional information examining associations between specific factors and delay were also extracted. A narrative synthesis was used to report the median range of diagnostic delay experienced by patients with axSpA and summarise which factors have a role in the delay. From an initial 11,995 articles, 69 reported an average time period of diagnostic delay, with 25 of these providing a median delay from symptom onset to diagnosis. Across these studies, delay ranged from 0.67 to 8 years, with over three-quarters reporting a median of between 2 years and 6 years. A third of all studies reported median delay data ranging from just 2 to 2.3 years. Of seven variables reported with sufficient frequency to evaluate, only ‘gender’ and ‘family history of axSpA’ had sufficient concordant data to draw any conclusion on their role, neither influenced the extent of the delay. Despite improvements in recent decades, patients with axSpA frequently experience years of diagnostic delay and this remains an extensive worldwide problem. This is further compounded by a mixed picture of the disease, patient and healthcare-related factors influencing delay.

**Table:** 

**Key points** *• Despite improvements in recent decades, patients with axSpA frequently experience years of diagnostic delay.* *• Median diagnostic delay typically ranges from 2 to 6 years globally.* *• Neither ‘gender’ nor ‘family history of axSpA’ influenced the extent of diagnostic delay experienced.* *• Diagnostic delay based on mean, rather than median, data influences the interpretation of the delay time period and consistently reports a longer delay period.*

**Supplementary Information:**

The online version contains supplementary material available at 10.1007/s10067-022-06100-7.

## Introduction

Axial spondyloarthritis (axSpA) is inflammatory arthritis characterised by inflammation in the sacroiliac joints (SIJ) and the axial spine, with symptoms such as chronic back pain, stiffness and fatigue most commonly manifesting in early adulthood [1]. In a 2017 UK representative primary care population, the incidence of axSpA was found to be 8.0 per 100,000 person-years and the prevalence 15.8 per 10,000 population [2].

The diagnosis of axSpA remains challenging due to the often-insidious onset of this condition, with initial presentation not always being immediately apparent as an inflammatory disease. Furthermore, although chronic back pain (CBP) lasting longer than three months is a key characteristic of axSpA, it frequently occurs in many patients with non-inflammatory back pain [3]. Hamilton et al. estimated that the prevalence of axSpA in the UK population of adults with CBP was only 1.3% [4], suggesting the vast majority of CBP in the general population is caused by conditions other than axSpA. Unfortunately, patients with axSpA commonly experience substantial delays before receiving a definitive diagnosis [5]. Such delay in the diagnosis of axSpA presents significant, multifaceted burdens to patients and society. Previous research has found that patients with axSpA who experience diagnostic delay have poorer general and disease-specific quality of life [6, 7], more progressive disease development [8], experience a reduced efficacy of disease-modifying medication [9] and report increased work disability [10, 11].

Despite these serious consequences, the factors associated with a delay in the diagnosis of axSpA and its true extent remain unclear. Although a recent 2021 systematic review and meta-analysis by Zhao et al. found the mean time period of delay in axSpA diagnosis to be 6.7 (6.2, 7.2) years globally and 8.6 (7.3, 10.0) years in the UK specifically, there remains a significant limitation with these estimates [12]. Diagnostic delay data is typically skewed by outliers, making the pooling of mean data less reliable and increasing the potential of an overestimation of the delay occurring in a population. The use of median data is recommended in the analysis of skewed data [5], and although this data cannot be pooled through meta-analysis to provide a convenient single average estimate of delay, a more accurate and reliable probable data range can be established. It also remains unclear whether specific patient-related factors, disease characteristics or healthcare systems processes have an influence on the extent of delay experienced by certain groups.

The aim of this systematic review was to ascertain the extent of, and potential reasons for, diagnostic delay in people with axSpA. Our two specific objectives were (1) to synthesise published literature detailing a median time period of delay from symptom onset to final diagnosis in patients with axSpA to determine a benchmark delay range and (2) to examine any factors associated with the extent of diagnostic delay experienced by patients with axSpA.

## Methods

A systematic review was conducted using five medical literature databases, and articles were searched for from journal inception to November 2021. These databases were Medline, EMBASE, AMED, CINAHL and Web of Science. The protocol was registered with PROSPERO in 2018 (registration number: CRD42019118963).

### Study selection

For studies to meet the inclusion criteria, several factors were required, with the primary outcome of interest, an average time period of diagnostic delay for axSpA (mean or median at inclusion stage). This also included any reporting of delay in time period subsets between symptom onset to final diagnosis (e.g., symptom onset to the first consultation, rather than the final diagnosis). Further inclusion criteria required that study types used cross-sectional, cohort, case-control or RCTs design of at least 20 adult participants. There were no restrictions on language, but those which could not be translated were not included in the systematic review. Additional to this search strategy, reference lists of existing reviews and included studies were reviewed to ensure the inclusion of any studies which may have been missed in the search strategy. Additionally, authors were contacted for any important missing data. Where further data was made available by authors, they are presented in this review as being linked with the published material.

Medical subject headings (MeSH) terms, or their database-specific equivalents, were searched, along with free-text phrases across all databases. The diagnostic/classification terminology of axial spondyloarthritis has evolved over time, and this was reflected in the search strategy created (Supplementary Table 1). Citations were exported into the reference management software Endnote X8, where duplicate articles were removed automatically. Using the specified inclusion criteria, articles were then initially screened by the first reviewer (CH) by title only, with any further de-duplication not achieved by Endnote being undertaken at this stage. After the review by title had been completed, a second reviewer (AC) was invited for the abstract review stage and the remaining articles were exported to Rayyan QCRI (https://www.rayyan.ai/) to facilitate a blinded review process based on abstract content. Following the abstract review stage, CH and AC both reviewed the remaining articles by full text. In the case of any disagreement between reviewers one and two, a third reviewer (JAP) arbitrated to reach a final decision. Finally, the reference lists of included studies were searched for further relevant studies.

### Data extraction and quality assessment

Data was extracted from all studies by a first reviewer (CH), with secondary data extraction being undertaken by a second (AC) and a third reviewer (JAP), each extracting data from half the included articles. Extracted data included the primary outcome of the time period of diagnostic delay, and study setting, sample size, country of origin, gender distribution of study population, method of diagnosis and any specific factors examined in relation to the experience of diagnostic delay. Methodological quality assessment was undertaken using the Newcastle-Ottawa (N-O) scale [13], with only questions which were deemed pertinent to this review retained. Quality assessment of all included studies was conducted independently by CAH and JAP.

### Analysis: extent of average diagnostic delay

A narrative synthesis was first undertaken to characterise extracted data. After extraction, for ease of comparison and interpretation across studies, reported time periods of diagnostic delay were standardised to number of years to two decimal places. Data were extracted from all studies which reported an average time period of diagnostic delay for axSpA, but for the purposes of this systematic review, those studies reporting median diagnostic delay were prioritised due to these being a potentially more accurate representation of the distribution of diagnostic delay. Finally, throughout the literature, both the terms ankylosing spondylitis and axial spondyloarthritis are used. While ankylosing spondylitis is now more commonly used as a synonym for radiographic-axSpA, it has historically been used to describe the entirety of axSpA and is still occasionally used synonymously with the entire spectrum of disease. Both terms were acceptable for inclusion, but to ascertain whether the terminology was related to trends in reported delay, a sensitivity analysis was undertaken by categorising reported delay by condition definition.

### Analysis: factors associated with diagnostic delay

Analysis on the extent of total diagnostic delay focused on median data. However, we did not impose this same restriction on articles which had examined differences in delay associated with specific factors (i.e. a family history of axSpA), and as such, different comparative approaches were taken to reflect different elements of delay. Firstly, to assess how the extent of diagnostic delay has changed over time, we compared articles reporting delay across multiple time-points in the same population. Secondly, to assess the role of a specific factor on delay required the primary outcome to be the existence of a comparison between the extent of delay between samples with or without a specific factor (e.g., comparison of diagnostic delay between men and women with axSpA). Here, our focus was on the existence of an examined difference between groups, and not the extent of the delay experienced. Where a specific factor of diagnostic delay was examined in five or more articles, the outcome of any statistical tests was recorded to examine whether a factor did, or did not, have a role in influencing the diagnostic delay experienced by patients with axSpA.

## Results

### Literature search

**Fig. 1:**
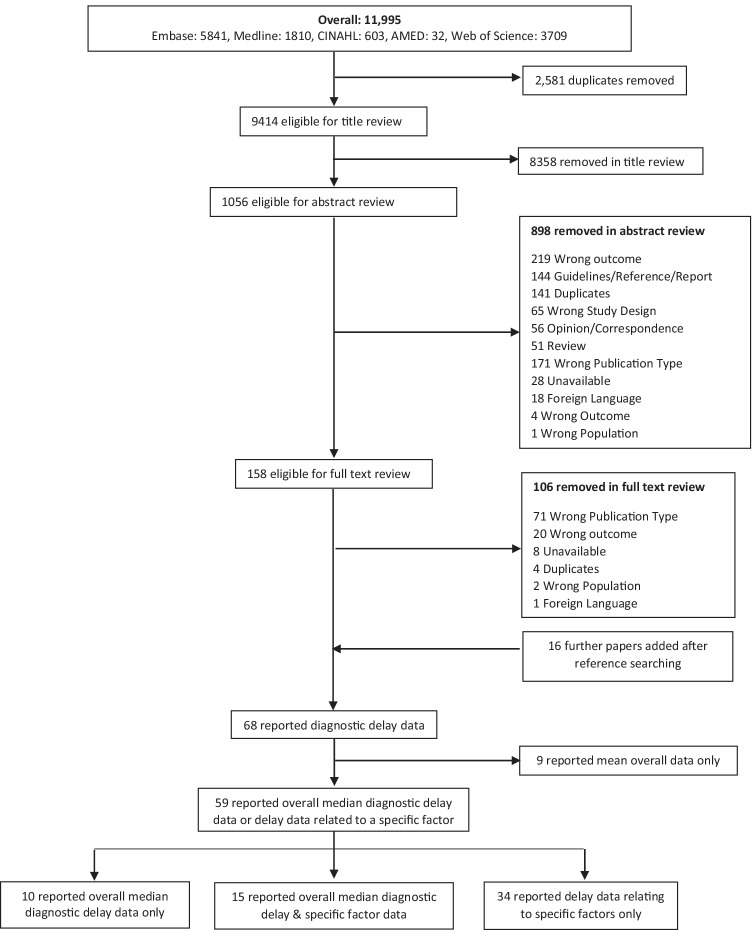
Flow diagram of the number of articles at each stage of the search and screening process

An initial 11,995 articles were identified, 2581 of which were duplicates; 8358 titles were excluded based on their title, leaving 1056 abstracts for review, followed by 158 articles to be reviewed in full. Finally, upon searching the references of the remaining articles, 16 more were found to be eligible for the systematic review, bringing the total number of articles included to 69. Of these, 59 reported either overall median diagnostic delay data or delay data related to a specific factor. This resulted in the identification of 25 individual median values which had specially examined the extent of diagnostic delay using the definition ‘from symptom onset to final diagnosis’ (Fig. 1). Finally, from the 34 articles which had examined the relationship between a specific factor and diagnostic delay in an axSpA sample, 4 had reported the change in delay over specific time periods.

### Extent of diagnostic delay

Twenty-five studies used the same definition, reporting median delay between the initial onset of axSpA symptoms and final diagnosis for the whole sample. Four studies were from Turkey [14–17], three from China [18–20], two each from Germany [21, 22], India [23, 24], South Korea [9, 25] and the UK [5, 26], and one each from the Czech Republic [27], Denmark [28], France [29], Iran [8], Italy [30], Norway [31], Saudi Arabia [32], Spain [33] and Thailand [34]. Garrido-Cumbrera et al. (2019) reported an overall diagnostic delay for Europe, including 13 countries (Austria, Belgium, France, Germany, Italy, the Netherlands, Norway, Russia, Slovenia, Spain, Sweden, Switzerland and the UK) [35]. The point at which average age was recorded across studies was not uniform and often recorded at multiple points, including at the time of the study, symptom onset or diagnosis. However, across these studies, the ‘average age at the time of the study’ ranged from the youngest at 32 years [20, 24] through to the oldest age of 55.9 years [22]. Where reported, males formed the majority of patients within all but one of the articles [35], with the percentage of males ranging from 52.9% [33] to 84.3% [23] (Table 1).

**Table 1:** Study characteristics for articles reporting median delay from symptom onset to axSpA diagnosis (*n* = 25)

Author	Year	Country	Sample size	Average age			Gender		Definition
				At time of study	At disease onset	At diagnosis	Male %	M:F	
Seo et al.	2014	South Korea	94	40 (IQR 39–49)	23 (IQR 17–30)	35 (IQR 24–43)	78.7	-	axSpA
Forejtova et al.	2008	Czech Republic	979	50.2 (SD 10.7)	27.3 (SD 8.5)	-	62.2	1.65:1	AS
Bakland et al.	2011	Norway	677	-	23.2 (SD 8.5)	-	-	3.1:1	AS
Fallahi et al.	2016	Iran	163	37.7 (SD 9.9)	23.4 (SD 7.1)	31.3 (SD 9.7)	79	-	AS
Hamilton et al.	2011	UK	807	-	-	-	-	3:1	AS
Merino et al.	2021	Spain	469	46.0 (SD 11.0)	23.8 (SD 8.5)	32.2 (SD 9.5)	52.9	-	axSpA
Aggarwal et al.	2009	India	70	-	23 (SD 8.8)	31.5 (SD 8.7)	84.3	5:1	AS
Brandt et al.	2007	Germany	350	40 (range 16–75)	-	-	-	-	axSpA
Sykes et al.	2015	UK	1193	-	-	-	-	-	axSpA
Gerdan et al.	2012	Turkey	393	39.3 (SD 10.8)	-	-	65.6	-	AS
Limsakul et al.	2021	Thailand	177	39.5 (SD 10.4)	28.5 (SD 9.6)	36.1 (SD 10.5)	62.1	-	axSpA
Garrido-Cumbrera et al.	2019	Europe	2846	43.9 (SD 12.3)	26.2 (SD 11.1)	33.7 (SD 11.5)	38.6	-	axSpA
Zengin et al.	2021	Turkey	308	36 (IQR 31–45)	-	31 (IQR 27–40)	58.8	-	axSpA
Ozgocmen et al.	2009	Turkey	279	36.11 (SD 10.2)	25.63 (SD 7.49)	30.7 (SD 9.42)	73	-	AS
Omair et al.	2017	Saudi Arabia	134	-	26 (IQR 20–33)	30 (25-38)	67.2	-	axSpA
Redeker et al.	2019	Germany	4471	55.9	30.6	-	54.1	-	axSpA
Li et al.	2019	China	208	35.5 (SD 12.8)	28.1 (SD 12.3)	-	71.6	-	axSpA
Salvadorini et al.	2012	Italy	135	-	28.3 (SD 10.2)	26.5 (SD 12.2)	-	90:45	axSpA
Bodur et al.	2010	Turkey	1381	39.5 (SD 10.7)	27.5 (SD 9.8)	32 (SD 10.7)	75.2	-	AS
Masson Behar et al.	2016	France	432	-	29.3 (SD 12.2)	34.2 (SD 12.5)	56.2	-	AS
Qian et al.	2017	China	1251	-	29.2 (11.4)	33.5 (12.6)	73.2	-	AS
Reddy et al.	2020	India	100	32 (IQR 26.0–36.7)	-	-	68	-	axSpA
Kong et al.	2021	China	270	32 (IQR 26–40)	22.5 (16–29)	-	78.9	-	AS
Hur et al.	2021	South Korea	1012	-	-	-	75.8	-	AS
Sorensen et al.	2014	Denmark	1335	40.8 (SD 12.4)	-	-	70.9	-	AS

The disease and diagnostic definitions were not standardised throughout the literature. Fourteen of the studies examining overall diagnostic delay described their patients as being diagnosed with ankylosing spondylitis (AS) [8, 14–16, 18, 20, 23, 25–29, 31, 33]. Ten of these [8, 14–16, 18, 20, 23, 25, 27, 31] defined AS using the modified New York Criteria (mNYC) [36], with one [29] using Assessment of Spondyloarthritis International Society (ASAS) criteria [37, 38]. The remaining three defined AS using International Classification of Diseases (ICD) codes [28], self-report [26] or through medical record [33]. Eleven studies described their patients as having axSpA [5, 9, 17, 19, 21, 22, 24, 29, 32, 34, 35]. Six of these [9, 17, 19, 24, 29, 34] classified axSpA using the ASAS criteria, while Brandt et al. used the mNYC [21], Sykes et al. and Merino et al. relied on physician verification [5, 33], Garrido-Cumbrera et al. relied on self-report [35] and Redeker et al. used ICD 10 codes [22].

Across the 25 studies, median diagnostic delay ranged from an average of 0.67 years in Denmark [28] to 8 years in South Korea [9]. Within this data range, though the one study reported a median delay of 0.67 years, over three-quarters of studies (80%) reported delay between 2 years and 6 years, with the articles reporting 2–2.3 years making up a third of the total (*n* = 8). A final three studies reported delay of 7 or 8 years (Table 2). When sensitivity analysis was undertaken by stratifying overall time periods of diagnostic delay based on disease definition, there was little variation in the range of delay reported for the different definitions (axSpA: 2–8 years vs. AS: 0.67–7.5 years) (Supplementary Table 2). Where mean delay data had also been reported alongside median data, each article consistently reported a greater mean delay. Across these 15 studies, the reporting of delay using mean data resulted in an average delay being 2.7 years longer than when median data was used (Supplementary Table 3).

**Table 2:** Extent of median delay in diagnosis of axSpA (*n* = 25)

Author	Year	Country	Sample size	Disease definition	Diagnostic delay(years)	IQR	Range
Seo et al.	2014	South Korea	94	axSpA	8	3–15	
Forejtova et al.	2008	Czech Republic	979	AS	7.5	3.5–12.5	
Bakland et al.	2011	Norway	677	AS	7		
Fallahi et al.	2016	Iran	163	AS	6		0–32
Hamilton et al.	2011	UK	807	AS	6	2–12	
Merino et al.	2021	Spain	469	AS	6	2–12	
Aggarwal et al.	2009	India	70	AS	5.9	3–11	
Brandt et al.	2007	Germany	350	axSpA	5		0.1–45
Sykes et al.	2015	UK	1193	axSpA	5	2–12	
Gerdan et al.	2012	Turkey	393	AS	5	11	
Limsakul et al.	2021	Thailand	177	axSpA	5	1.7–11.1	
Garrido-Cumbrera et al.	2019	Europe	2846	axSpA	4		
Zengin et al.	2021	Turkey	308	axSpA	4	3–5.5	
Ozgocmen et al.	2009	Turkey	279	AS	3		
Omair et al.	2017	Saudi Arabia	134	axSpA	3	1–6	
Redeker et al.	2019	Germany	4471	axSpA	2.3		0.1–7.2
Li et al.	2019	China	208	axSpA	2.1		4–74.8
Salvadorini et al.	2012	Italy	135	AS	2.1		2–3
Bodur et al.	2010	Turkey	1381	AS	2		
Masson Behar et al.	2016	France	432	axSpA	2	1–7	
Qian et al.	2017	China	1251	AS	2	0–2	
Reddy et al.	2020	India	100	axSpA	2	0.5–5	
Kong et al.	2021	China	270	AS	2	0–5	
Hur et al.	2021	South Korea	1012	AS	1	0.25–4	
Sorensen et al.	2014	Denmark	1335	AS	0.67		

### Extent of diagnostic delay over time

Four studies reported the change in diagnostic delay over time. Salvadorini et al. (2012) presented median delays over six decades from the 1950s through to 2000, showing a reduction in delay, with this halving every decade, apart from the 1970s to 1980s [30]. Calin et al. (1988) presented median delay over 15 time periods from the UK and also compared male delay with female delay over the same period [39]. This study found a reduction in delay throughout the 20th century, although not to the extent found by Salvadorini et al.; overall median diagnostic delay in the UK in the middle of the 20th century was far shorter than in Italy. Reed et al.’s Australian study (2008) presented mean diagnostic delay over three decades, from 1978 to 1993, which showed diagnostic delay reducing from 13.8 years in 1978 to 4.3 years in 1993 [40]. Finally, a US study by Wright et al. (2015) examined the extent of a diagnostic delay from 1980 through to 2009. Though a reduction in delay was found, this was minimal, with delay from 1980 to 1999 being 6.2 years, and between 2000 and 2009 being 5.6 years [41].

### Factors association with diagnostic delay

Forty-five studies [5, 8, 10, 14–19, 21, 23, 24, 27, 32, 34, 42–71] examined the role of at least one specific factor for a possible association with diagnostic delay. In total, these studies reported 47 distinct factors, which were separated into 16 categories (Supplementary Tables 4 and 5). The majority of these were only investigated once in a single study, but seven factors had been statistically compared in more than five separate studies. These factors included gender (20 studies comparing delay between men and women [5, 8, 14, 17–19, 23, 24, 42–52]), HLA-B27 negativity vs positivity (15 studies [8, 17–19, 23, 32, 34, 42, 44, 45, 49, 59, 60, 65, 71]), radiographic- vs. non-radiographic-axSpA (9 studies [19, 21, 49, 54–56, 67, 68, 72]), juvenile vs adult disease-onset (7 studies [8, 14, 16, 18, 19, 23, 49]), family history of axSpA (5 studies [8, 19, 23, 42, 45]), presence at onset of uveitis or not (5 studies [5, 8, 19, 44, 45]) and presence of peripheral arthritis or not (5 studies [5, 8, 23, 45, 49]). Of these factors, gender and family history of axSpA had sufficient data concordance to determine that they had no influence on the extent of diagnostic delay experienced across the majority of studies. The study findings for the remaining factors reported contradictory directions of effect, preventing the determination of any definite association (or lack thereof) (Table 3).

**Table 3:** Summary of factors and their association on diagnostic delay in axSpA

	Characteristics	Total no of studies comparing factor	Decreased delay	No difference	Increased delay
Directional impact on delay	Gender *(male)*	18	1	15	2
	Family history of axSpA *(yes)*	5	0	5	0
Mixed impact on delay	HLA-B27 *(+)*	15	7	6	2
	Radiographic axSpA (*yes*)	9	0	5	4
	Age of onset *(<16 years)*	5	0	3	2
	Peripheral arthritis *(yes)*	5	1	3	1
	Uveitis *(yes)*	5	1	3	1

Of the 20 studies which examined the difference in the extent of diagnostic delay experienced between men and women, 18 had undertaken a statistical comparison, of which only three found a significant difference in the delay of receiving a diagnosis between the sexes. With one exception [48], all of these studies had a greater proportion of men in their sample, ranging from 52.3 to 92.7%. The majority of samples (88.9%) were recruited from secondary care health settings, the exceptions being two studies, one using the general population [43] and another using patients from a defence medical rehabilitation centre [47]. Just under two-thirds (60.0%) of articles defined disease as AS rather than axSpA (Table 4).

**Table 4:** Articles comparing diagnostic delay between (i) males and females (*n* = 20) and (ii) patients with or without a family history of AS/axSpA (*n* = 5)

Author	Year	Country	Sample size	Disease definition	Male (%)	Extent of diagnostic delay (years)					
						By gender			By family history		
						Males	Female	*P*-values	Yes	No	*P*-values
Bandinelli et al.	2016	Italy	135	axSpA	67.4	9.91	6.3	**0.0023**	9.48	8.68	0.55
Geirsson et al.	2010	Iceland	223	AS	65	8.3	9.6	0.87	-	-	-
Nakashima et al.	2015	Japan	72	AS	83	6.9	5.5	0.47	-	-	-
Ma et al.	2012	South China	70	AS	72.9	6.6	6.2	N/S	-	-	-
Aggarwal et al.	2009	India	70	AS	84.3	6.5	8.6	0.23	7.1	6.6	0.68
Fallahi et al.*	2016	Iran	163	AS	79	6	6.5	0.68	6.5	6	0.32
Hajialilo et al.	2014	Iran	60	AS	88.3	5.9	8	0.14	6.5	6	0.64
Slobodin et al.	2010	Israel	151	axSpA	52.3	5.9	5.7	0.87	-	-	-
Roussou et al.	2010	UK	516	axSpA	33.3	5.56	6.27	N/S	-	-	-
Jones et al.	2014	UK	138	axSpA	-	5.56	8.5	-	-	-	-
Dincer et al.	2007	Turkey	111	AS	92.7	5.32	14.42	0.061	-	-	-
Sykes et al.*	2015	UK	1193	axSpA	-	5	6	N/S	-	-	-
Coughlan et al.	1981	Ireland	78	AS	73	4.6	5	-	-	-	-
Ibn Yacoub et al.	2012	Morocco	130	AS	66.9	4.6	4.8	0.075	-	-	-
Ma et al.	2012	North China	80	AS	78.6	4	4.1	N/S	-	-	-
Zengin et al.*	2021	Turkey	308	axSpA	58.8	4	4	0.238	-	-	-
Li et al.*	2019	China	208	axSpA	71.6	2.92	1.04	**0.014**	1.36	2.38	N/S
Bodur et al.*	2012	Turkey	1381	AS	75.2	2	2.3	0.385	-	-	-
Qian et al.*	2017	China	1251	AS	73	2	2	N/S	-	-	-
Reddy et al.*	2020	India	100	axSpA	68	1	4	**0.021**	-	-	-

*Diagnostic delay reported as median

*P*-values in **bold** indicates statistical significance. Dash (-) indicates no test for significance performed

*AS*, ankylosing spondylitis; *AxSpA*, axial spondyloarthritis; *UK*, United Kingdom; *N*/*S*, non-significant

Five articles had assessed the role that patients’ family history of axSpA has on the extent of diagnostic delay experienced. All articles used samples from secondary care, were predominantly male (ranging from 67.4 to 84.3%) and found no association between the delay experienced in receiving a delayed diagnosis and whether patients did, or did not, have a family history of the same condition. Three out of five studies defined their disease of interest as AS rather than axSpA (Table 4).

### Quality assessment

Of the 59 included studies, 28 had samples considered truly representative of the population of patients with axSpA, while 29 were somewhat representative of the population. Two had samples from a selected group. Ascertainment of exposure, i.e., proof of diagnosis, was made using secure records in 43 studies (the highest standard of quality for this item), while 11 were ascertained using structured interviews. Written self-report was used in three studies, and two did not describe how they ascertained patients’ diagnosis. Assessment of outcome, i.e., assessment of diagnostic delay, did not reach the highest standard (independent blind assessment) in any study. Record linkage was used in 23 studies, and a further 28 used patient self-report. Eight did not describe how the diagnostic delay was confirmed. Ascertainment of exposure was the criteria most commonly fulfilled to the highest criteria (*n* = 43).

## Discussion

This systematic review collated all available studies examining the extent of diagnostic delay in patients with axSpA, prioritising those reporting median data. Across all countries, patients with axSpA are experiencing years of diagnostic delay, the majority between 2 and 6 years, though delay appears to have reduced somewhat over the second half of the 20th century. Our findings also highlight the extent to which diagnostic delay based on mean data, rather than median, influences the interpretation of delay, with such studies consistently reporting longer periods of delay. We also found that many disease-, patient- or healthcare-related factors had been considered in relation to their role on the extent of diagnostic delay experienced by patients with axSpA, but that few of these factors were examined across multiple studies, making it difficult to evaluate their part in delay. Where the role of a factor had been sufficiently examined, many articles reported contradictory directions of effect. However, ‘family history of axSpA’ and ‘gender’ were more concordant, showing no significant role in the extent of diagnostic delay experienced by patients.

### Extent of diagnostic delay

It is evident that a patient with axSpA will most likely be delayed in receiving their diagnosis from between 2 and 6 years. Across this collection of studies, we were unable to identify any variables which influenced the length of delay reported, and this 4-year range could relate to the inclusion of studies with different sample sizes and health care settings. Even within the same country, delay ranged across several years, as exemplified by multiple studies from the UK (5–6 years), Turkey (2–5 years) and Germany (2.3–5 years). However, despite this, the range of 2–6 years does provide the axSpA community with a clearer benchmark of the current problem of delay across the globe. Our proposal that this range is ‘typical’ is further strengthened by the included article by Garrido-Cumbrera et al. [35]. Their large study of diagnostic delay across 13 European countries found a median delay of 4 years, falling midway within our own range. Furthermore, and somewhat encouragingly, a third (*n* = 8) of all articles reported a median delay of 2–2.3 years. Our findings were not influenced by the use of the different disease terms of ‘AS’ and ‘axSpA’, despite the fact that ‘AS’ would generally have been used before the ‘axSpA’ classification criteria was adopted.

Though the recent review by Zhao et al. contained twelve of the same articles identified within our assessment of median data [5, 8, 9, 15, 16, 23, 26–29, 31, 35], their focus on mean data makes direct comparisons with our work difficult. We would suggest that our findings represent a more accurate indication of the ‘usual’ patient journey from symptom onset to final diagnosis due to the use of median data. The pooled diagnostic delay of 6.7 years (95% CI 6.2–7.2) found by Zhao et al. exceeds the average reported by the majority of studies our review identified. Furthermore, where both measures of central tendency had been reported in the same study, this ‘overestimation’ of mean delay was consistently demonstrated. Finally, the argument for the use of median, over mean diagnostic delay data in patients with axSpA has been made previously by Sykes et al. (2015). Their study showed a median delay of 5.0 years, compared to a mean delay of the same sample of 8.5 years in a UK population [5]. Furthermore, they found that a third of patients experienced less than two years delay, and over a half experienced less than five years delay, results consistent with our own.

The results presented by our review provide a new, more accurate benchmark for general and country-specific diagnostic delay against which to measure success regarding the speed of diagnosis. Such benchmarks need to be as accurate and representative of the greatest number of patients as possible. Any concern about the loss of representation of the extremes of diagnostic delay can be allayed through presenting ranges associated with delay medians. It is therefore important that, when discussing diagnostic delay in axSpA, medians are the prioritised result. Such information from our review is needed as a starting point, so that the medical community can work towards the concept of a limited window of opportunity to achieve a swift diagnosis, prompt treatment and management provision for patients, as now achieved in rheumatoid arthritis [73].

### Role of specific factors on diagnostic delay experience

This review was able to consider how diagnostic delay for axSpA has changed over time. Despite the limited number of articles examining this concept, since the mid-20th century, it appears that diagnostic delay has been reducing. Reasons for this may be related to increased disease awareness [30], advances in diagnostic technology, such as magnetic resonance imaging [74] and improved aetiological understanding [75]. However, in recent decades, there are some suggestions that the rate of reduction of delay may be plateauing, implying further policy and methodological change is required to continue reduction to delay.

Of all the factors which have previously been examined in relation to their role on delay, only ‘gender’ and ‘family history of axSpA’ were studied to a sufficient extent and reported enough concordant findings to be able to draw conclusions on their role upon delay. The predominance of non-significant findings across the vast majority of these studies suggests that neither factors have a role in the delay experienced by patients with axSpA. The historical misconception of axSpA as a ‘male disease’ would suggest that delay should be greater in females. However, this has not been translated into the findings from the studies which have specifically compared the diagnostic delay between men and women. This could relate to sample selection issues, but the high frequency of studies found would suggest this is a consistent event.

Five studies examined the relationship between family history of axSpA and diagnostic delay, of which none found a significant association. In contrast, having a family member with axSpA has previously been shown to be a risk factor for developing axSpA. Lunteran et al. reported 15% of patients suspected of axSpA having a family history of the disease, and a strong association between a patient having first-degree relatives with AS and being positive for HLA-B27 (OR 7.8 (95% CI 3.8-16.0) [76]. Ez-Zaitouni et al. also found a positive association between family history and ASAS criteria in two cohorts (OR 3.3 (95% CI 2.0–5.3); OR 2.1 (1.3–3.3), respectively) [77]. It is therefore plausible that a family history of axSpA is a factor which would reduce diagnostic delay in patients presenting with axSpA-like symptoms, potentially due to patients’ increased knowledge of the condition or healthcare professionals being aware of an increased risk of axSpA development in individuals with a spondyloarthritis family history. However, the fact that patients with a family history of axSpA appear to experience no difference in delay than patients with no family history of the disease could be the result of several factors. The disparate symptom profiles may not register with the already diagnosed family member, and lack of contact or an unwillingness for individuals to share health problems with family members may also be factors. Though Ez-Zaitouni et al. found family history to be associated with ASAS criteria for axSpA, it was not associated with sacroiliitis [77]. The implication here could be that patients’ symptoms, while diagnostically suggestive, might not be similar enough to their relatives’ symptoms for them to raise in consultation with their healthcare professional. It could also be that in primary care, the question of family history does not arise early on and only arises after the healthcare professional being consulted had become suspicious of the disease. All these issues suggest education and discussion of health (particularly hereditary) conditions should be promoted in patients with axSpA.

### Strengths and limitations

The main strength of this systematic review is that it is the first to synthesise the median delay data to provide a global benchmark range, presenting more robust delay values across individual countries, and providing the most accurate understanding of diagnostic delay for axial spondyloarthritis to date. This is reinforced by the direct comparison of means to medians in studies where they are calculated from the same sample. Additionally, this systematic review details and compares many disease-, patient- and healthcare-related factors which are associated with diagnostic delay, which suggests several avenues for future research. The main limitation of this systematic review is the high level of variability, even within the same countries, which is apparent across the included studies.

## Conclusion

This systematic review has highlighted that, despite marked improvements over the last few decades, the delay experienced by patients in receiving a diagnosis of axial spondyloarthritis remains unacceptably long across many counties. However, our focus on median data does provide a more robust indicator of the extent of this delay for the majority of patients and can therefore act as a new benchmark for future research. Regarding the role of disease and patient-related factors, gender and family history do not appear to influence diagnostic delay, but while studies examining other factors were numerous, evidence of associations between patient characteristics and diagnostic delay remain contradictory or limited. Healthcare systems must continue to strive to reduce the delay experienced by patients, and further rigorous research examining which patient groups are most vulnerable to experiencing delay is needed.

## Supplementary Information

ESM 1 (DOCX 103 kb)
