# Supplementary material for: Diagnostic delay in axial spondyloarthritis: a systematic review
Source: Clin Rheumatol. 2022 Feb 19;41(7):1939–50. doi: 10.1007/s10067-022-06100-7 (PMC9187558; doi:10.1007/s10067-022-06100-7)
Supplement: Supplementary file 1 — (DOCX 103 kb) [file 10067_2022_6100_MOESM1_ESM.docx]

Supplementary table 1: Search criteria

| # | Search Term |
| --- | --- |
| 1 | Ankylosing ADJ spondyl*.ti,ab,kw |
| 2 | Spondylitis, ankylosing (MeSH) |
| 3 | Spondyloarth*.ti,ab,kw |
| 4 | Spondylarth*.ti,ab,kw |
| 5 | Spondylitis.ti,ab,kw |
| 6 | Spondylarthritis (MeSH) |
| 7 | Spondylarthropathies (MeSH) |
| 8 | Spondylitis (MeSH) |
| 9 | Bechtere*.ti,ab,kw |
| 10 | Marie-str*.ti,ab,kw |
| 11 | (Bamboo ADJ spine).ti,ab,kw |
| 12 | (Spin* ADJ3 Arthr*).ti,ab,kw |
| 13 | Sacroil*.ti,ab,kw |
| 14 | Sacroiliitis (MeSH) |
| 15 | 1 or 2 or 3 or 4 or 5 or 6 or 7 or 8 or 9 or 10 or 11 or 12 or 13 or 14 |
| 16 | Delayed Diagnosis (MeSH) |
| 17 | Early Diagnosis (MeSH) |
| 18 | ((late* or earl*) ADJ3 diagnos*).ti,ab,kw |
| 19 | ((late* or earl*) ADJ3 treat*).ti,ab,kw |
| 20 | ((late* or earl*) ADJ3 consult*).ti,ab,kw |
| 21 | ((late* or earl*) ADJ3 refer*).ti,ab,kw |
| 22 | ((late* or earl*) ADJ3 detect*).ti,ab,kw |
| 23 | (diagnos* ADJ3 delay*).ti,ab,kw |
| 24 | (diagnos* ADJ3 lag*).ti,ab,kw |
| 25 | (diagnos* ADJ3 interval*).ti,ab,kw |
| 26 | (treatment* ADJ3 delay*).ti,ab,kw |
| 27 | (case* ADJ3 find*) .ti,ab,kw |
| 28 | (case* ADJ3 seek*).ti,ab,kw |
| 29 | (health* ADJ3 seek*) .ti,ab,kw |
| 30 | (care ADJ3 seek*) .ti,ab,kw |
| 31 | (delay* adj3 consult*).ti,ab,kw |
| 32 | (delay* adj3 detect*).ti,ab,kw |
| 33 | (delay* adj3 interval*).ti,ab,kw |
| 34 | (Delay* adj3 refer*).ti,ab,kw |
| 35 | (delay* adj3 seek*).ti,ab,kw |
| 36 | 16 or 17 or 18 or 19 or 20 or 21 or 22 or 23 or 24 or 25 or 26 or 27 or 28 or 29 or 30 or 31 or 32 or 33 or 34 or 35 |
| 37 | 15 and 36 |

Supplementary table 2: Sensitivity analysis comparing delay by disease definition

| Author | Year | Median delay (years) |
| --- | --- | --- |
| **axSpA (n=11)** | | |
| Seo at al | 2014 | 8 |
| Brandt et al | 2007 | 5 |
| Sykes et al | 2015 | 5 |
| Limsakul et al | 2021 | 5 |
| Garrido-Cumbrera et al | 2019 | 4 |
| Zengin et al | 2021 | 4 |
| Omair et al | 2017 | 3 |
| Redeker et al | 2019 | 2.3 |
| Li et al | 2019 | 2.1 |
| Salvadorini et al | 2012 | 2.1 |
| Reddy et al | 2022 | 2 |
| **AS (n=14)** | | |
| Forejtova et al | 2008 | 7.5 |
| Bakland et al | 2011 | 7 |
| Fallahi et al | 2016 | 6 |
| Hamilton et al | 2011 | 6 |
| Merino et al | 2021 | 6 |
| Aggarwal et al | 2009 | 5.9 |
| Gerdan et al | 2012 | 5 |
| Ozgocmen et al | 2009 | 3 |
| Bodur et al | 2010 | 2 |
| Masson Behar et al | 2016 | 2 |
| Qian et al | 2017 | 2 |
| Kong et al | 2022 | 2 |
| Hur et al | 2021 | 1 |
| Sorensen et al | 2014 | 0.67 |

Supplementary table 3: Difference in time-period of diagnostic delay in studies reporting both mean and median data (n=15)

|  |  |  | **Diagnostic delay** | |  |
| --- | --- | --- | --- | --- | --- |
| **Author** | **Year** | **Country** | **Mean** | **Median** | **Difference in delay estimate**  **(Mean-Median) years** |
| Forejtova et al | 2008 | Czech Republic | 9.1 | 7.5 | 1.7 |
| Bakland et al | 2011 | Norway | 9 | 7 | 2 |
| Fallahi et al | 2016 | Iran | 7.88 | 6 | 1.88 |
| Hamilton et al | 2011 | UK | 8.57 | 6 | 2.57 |
| Aggarwal et al | 2009 | India | 6.9 | 5.9 | 1 |
| Brandt et al | 2007 | Germany | 7.7 | 5 | 2.7 |
| Gerdan et al | 2012 | Turkey | 8.12 | 5 | 3.12 |
| Sykes et al | 2015 | UK | 8.53 | 5 | 3.53 |
| Garrido-Cumbrera et al | 2019 | Europe | 7.4 | 4 | 3.4 |
| Ozgocmen et al | 2009 | Turkey | 5.08 | 3 | 2.08 |
| Redeker et al | 2019 | Germany | 5.7 | 2.3 | 3.4 |
| Li et al | 2019 | China | 4.83 | 2.13 | 2.7 |
| Behar et al | 2016 | France | 4.9 | 2 | 2.9 |
| Bodur et al | 2010 | Turkey | 5 | 2 | 3 |
| Sorensen et al | 2014 | Denmark | 5.25 | 0.67 | 4.58 |
| Mean combined difference in delay |  |  |  |  | 2.7 |

Supplementary table 4: Factors examined in relation to their role on the extent of diagnostic delay experienced

| **Factor categories (n=16)** | **Specific factors examined (n=47)** |
| --- | --- |
| Gender | Gender |
| Race | Race |
| Geographical region | Geographical region |
| Referral process | Direct rheumatology referral |
|  | Initial visit to a rheumatologist |
| Diagnostician | Expert rheumatologist |
| Disease type | Radiographic axSpA |
| Patient history | Family history of axSpA |
|  | History of nephrolithiasis |
|  | History of infection prior to disease |
|  | History of smoking |
|  | 1st degree, Sero-SpA relative |
|  | History of infection prior to diagnosis |
|  | Prior diagnosis of lumbar disc herniation |
| Age of disease onset | less than 16 years |
|  | Juvenile onset |
|  | less than 20 years |
|  | less than 45 years |
|  | Juvenile onset |
|  | Year of onset before 1999 |
| Symptoms | Peripheral arthritis |
|  | Enthesitis |
|  | Uveitis |
|  | Articular involvement |
|  | Extra-articular involvement |
|  | Spinal initial symptoms |
|  | Inflammatory back pain at onset |
|  | Radiological sacroiliitis at onset |
|  | Sacroiliitis radiological stage 1-2 |
|  | Minimal hip disease |
|  | Buttock pain |
|  | Morning stiffness |
|  | Sleep |
| Comorbidity | Inflammatory bowel disease |
|  | Comorbidity |
|  | Psoriasis |
| HLA-B27 | HLA-B27+ |
| Clinical signs | ESR >30 |
|  | CRP >6 |
|  | Anti-TNF |
| Education | Less than 9 years education |
|  | Low education |
|  | 0-8 years education |
| Employment | Full disability pension |
|  | Manual employment |
|  | Work disability |
|  | Changed work |

Supplementary table 5: Diagnostic delay associated with specific factors

| **Characteristics** | **Author** | **Year** | **Extent of diagnostic delay by characteristic (Years)** | | |  | **P-values** |
| --- | --- | --- | --- | --- | --- | --- | --- |
| Race |  |  | *Arab* | *Caucasian* | *Indian sub-continent* |  |  |
|  | Quraishi et al | 2018 | 2.89 | 1.87 | 3.85 |  | 0.39 |
| Region |  |  | *South China* | *North China* |  |  |  |
|  | **Ma et al** | **2012** | **7.3** | **3.2** |  |  | **<0.0001** |
| Referral process |  |  | Direct rheumatology referral | 1st referral non-rheumatology |  |  |  |
|  | Kidd et al* | 1988 | 3 | 6 |  |  | - |
|  |  |  | *Initial visit non-rheumatologist* | *Initial visit rheumatologist* |  |  |  |
|  | **Li et al*** | **2019** | **2.54** | **0.54** |  |  | **0.018** |
| Diagnostician |  |  | Expert rheumatologist | General rheumatologist | GP |  |  |
|  | Roussou et al* | 2011 | 6 | 10 | >15 |  | - |
| Disease type |  |  | *Radiographic axSpA* | *Non-radiographic axSpA* |  |  |  |
|  | Kidd et al* | 1988 | 3 | 2.83 |  |  | - |
|  | Brandt et al* | 2007 | 8 | 2 |  |  | - |
|  | **Li et al*** | **2019** | **3.04** | **0.5** |  |  | **<0.0001** |
|  | Kishimoto et al* | 2021 | 2.8 | 1.9 |  |  | 0.14 |
|  | Burgos-Varga et al | 2016 | 6.48 | 5.21 |  |  | 0.747 |
|  | **Gavali et al** | **2015** | **4.4** | **1.3** |  |  | **<0.0001** |
|  | **Chimenti et al** | **2019** | **5.91** | **3.04** |  |  | **0.007** |
|  | Dincer et al | 2007 | 6.63 | 5.53 |  |  | 0.407 |
|  |  |  | *Radiographic axSpA* | *Non-radiographic axSpA MRI arm* | *Non-radiographic axSpA clinical arm* |  |  |
|  | **Su et al*** | **2021** | **0.5** | **0.58** | **0.17** |  | **0.008** |
| History |  |  | *History of nephrolithiasis* | *No history of nephrolithiasis* |  |  |  |
|  | Fallahi et al* | 2016 | 5 | 6 |  |  | 0.44 |
|  |  |  | *History of infection prior to disease* | *History of infection prior to disease* |  |  |  |
|  | Fallahi et al* | 2016 | 3 | 6 |  |  | 0.31 |
|  |  |  | *History of smoking* | *No history of smoking* |  |  |  |
|  | **Li et al*** | **2019** | **3.04** | **1.58** |  |  | **0.043** |
|  |  |  | *1st degree, Sero- SpA relative* | *No 1st degree, Sero- SpA relative* |  |  |  |
|  | **Dincer et al** | **2007** | **4.6** | **10** |  |  | **0.003** |
|  |  |  | *Prior diagnosis of lumbar disc herniation* | *No prior diagnosis of lumbar disc herniation* |  |  |  |
|  | **Gerdan et al** | **2012** | **9.1** | **6.2** |  |  | **0.002** |
|  |  |  | *Smoker* | *Non-smoker* |  |  |  |
|  | Bedaiwi et al | 2021 | 6.31 (6.86) | 7.04 (5.72) |  |  |  |
| Age of onset |  |  | *<16 yrs* | *>16 yrs* |  |  |  |
|  | Fallahi et al* | 2016 | 5.5 | 6 |  |  | 0.91 |
|  | Qian et al* | 2017 | 2 | 2 |  |  | N/S |
|  | Li et al* | 2019 | 2.17 | 2.13 |  |  | N/S |
|  | **Dincer et al** | **2007** | **8.89** | **5.51** |  |  | **0.027** |
|  | **Aggarwal et al** | **2009** | **9.1** | **6.1** |  |  | **0.03** |
|  |  |  | *Juvenile onset* | *Adult onset* |  |  |  |
|  | **Ozgocmen et al*** | **2009** | **9** | **3** |  |  | **<0.001** |
|  | **Bodur et al** | **2010** | **7.6** | **4.7** |  |  | **<0.001** |
|  |  |  | *<20yrs* | *>20yrs* |  |  |  |
|  | Nakashima et al | 2015 | 7 | 6.4 |  |  | 0.17 |
|  |  |  | *<45 years* | *>45 years* |  |  |  |
|  | **Zwolak et al** | **2019** | **6.2** | **18** |  |  | **<0.0001** |
|  |  |  | Early onset (<50 years) | Late onset (≥50 years) |  |  |  |
|  | **Maatallah et al** | **2021** | **4** | **2** |  |  | **0.05** |
| Year of onset |  |  | *<1999* | *>2000* |  |  |  |
|  | **Nakashima et al** | 2015 | **7.5** | **2.6** |  |  | **0.02** |
| Symptoms |  |  | Peripheral arthritis | No Peripheral Arthritis |  |  |  |
|  | **Sykes et al*** | **2015** | **4** | **6** |  |  | **0.025** |
|  | Fallahi et al* | 2016 | 6 | 5 |  |  | 0.086 |
|  | **Hajialilo et al** | **2014** | **11.3** | **5.1** |  |  | **0.0001** |
|  | Aggarwal et al | 2009 | 6.8 | 6.4 |  |  | 0.8 |
|  | Dincer et al | 2007 | 4.78 | 6.55 |  |  | 0.291 |
|  |  |  | *Enthesitis* | *No enthesitis* |  |  |  |
|  | **Fallahi et al*** | **2016** | **6** | **4** |  |  | **0.007** |
|  | **Hajialilo et al** | **2014** | **13** | **5.9** |  |  | **0.004** |
|  |  |  | *Uveitis* | *No uveitis* |  |  |  |
|  | Fallahi et al* | 2016 | 5 | 6 |  |  | 0.71 |
|  | Li et al* | 2019 | 3.61 | 1.9 |  |  | N/S |
|  | **Sykes et al*** | **2015** | **10** | **5** |  |  | **0.005** |
|  | Nakashima et al | 2015 | 7.5 | 6.5 |  |  | 0.86 |
|  | **Hajialilo et al** | **2014** | **2.4** | **6.4** |  |  | **0.02** |
|  |  |  | *Articular involvement* | *No articular involvement* |  |  |  |
|  | **Nakashima et al** | **2015** | **5.2** | **8.9** |  |  | **0.03** |
|  |  |  | *Extra-articular involvement* | *No extra-articular involvement* |  |  |  |
|  | **Aggarwal et al** | **2009** | **8.7** | **5.9** |  |  | **0.03** |
|  |  |  | *Spinal initial symptoms* | *Extra-spinal initial symptoms* |  |  |  |
|  |  |  | 7 | 6.4 |  |  | 0.5 |
|  |  |  | *Inflammatory back* | *No Inflammatory* |  |  |  |
|  |  |  | *pain at onset* | *back pain at onset* |  |  |  |
|  | Aggarwal et al | 2009 | 7.3 | 5.9 |  |  | 0.3 |
|  | **Hajialilo et al** | **2014** | **4.8** | **8.7** |  |  | **0.001** |
|  | **Dincer et al** | **2007** | **3.28** | **8.57** |  |  | **0.001** |
|  |  |  | *Radiological* | *No Radiological* |  |  |  |
|  |  |  | *sacroiliitis at onset* | *sacroiliitis at onset* |  |  |  |
|  | Dincer et al | 2007 | 6.63 | 5.53 |  |  | 0.407 |
|  |  |  | *Sacroiliitis radiological stage 1-2* | *Sacroiliitis radiological stage 3-5* |  |  |  |
|  | Koko et al | 2014 | 1.6 | 3.3 |  |  | 0.021 |
|  |  |  | *Minimal hip disease* | *Moderate hip disease* | *Severe hip disease* |  |  |
|  | **Zhao et al** | 2015 | **3.46** | **3.68** | **4.59** |  | **0.001** |
|  |  |  | *Buttock pain* | *No buttock pain* |  |  |  |
|  | Hajialilo et al | 2014 | 5.3 | 7 |  |  | 0.07 |
|  |  |  | *Morning stiffness* | *No morning stiffness* |  |  |  |
|  | Dincer et al | 2007 | 7.29 | 5.16 |  |  | 0.174 |
|  | **Hajialilo et al** | **2014** | **4.6** | **10.1** |  |  | **0.0001** |
| Comorbidity |  |  | *IBD* | *No IBD* |  |  |  |
|  | **Sykes et al*** | **2015** | **4** | **6** |  |  | **0.024** |
|  | Fallahi et al* | 2016 | 8 | 6 |  |  | 0.87 |
|  | Nakashima et al | 2015 | 7.9 | 6.5 |  |  | 0.2 |
|  |  |  | *Comorbidity* | *No comorbidity* |  |  |  |
|  | Nakashima et al | 2015 | 8.3 | 5.6 |  |  | 0.24 |
|  |  |  | *Psoriasis* | *No psoriasis* |  |  |  |
|  | Nakashima et al | 2015 | 8.3 | 6.4 |  |  | 0.57 |
| HLA-B27 |  |  | *HLA-B27+* | *HLA-B27-* |  |  |  |
|  | **Fallahi et al*** | **2016** | **5** | **9** |  |  | **0.013** |
|  | Qian et al* | 2017 | 2 | 2 |  |  | N/S |
|  | Omair et al* | 2017 | 4 | 2 |  |  | 0.186 |
|  | **Zengin et al*** | **2021** | **3.7** | **5** |  |  | **0.015** |
|  | **Li et al*** | **2019** | **2.79** | **0.5** |  |  | **0.009** |
|  | **Zhang et al*** | **2020** | **1** | **1.5** |  |  | **0.034** |
|  | **Limsakul et al*** | **2021** | **6** | **2.5** |  |  | **0.001** |
|  | **Feldtkeller et al** | **2003** | **8.5** | **11.4** |  |  | **<0.001** |
|  | **Dincer et al** | **2007** | **5.33** | **9.20** |  |  | **0.037** |
|  | Bandinelli et al | 2016 | 8.433 | 10.26 |  |  | 0.3446 |
|  | Bakland et al | 2005 | 7.9 | 8.5 |  |  | 0.9 |
|  | Aggarwal et al | 2009 | 6.9 | 6.6 |  |  | 0.9 |
|  | Nakashima et al | 2015 | 6.6 | 6 |  |  | 0.84 |
|  | **Hajialilo et al** | **2014** | **4.6** | **10.1** |  |  | **0.0001** |
|  | **Chung et al** | **2011** | **2.7** | **3.7** |  |  | **0.01** |
| Other Clinical Signs |  |  | *ESR >30* | *ESR <30* |  |  |  |
|  | **Hajialilo et al** | **2014** | **4.8** | **7.9** |  |  | **0.0001** |
|  |  |  | *CRP >6* | *CRP <6* |  |  |  |
|  |  |  | **5.6** | **7.8** |  |  | **0.036** |
| Treatment |  |  | *Anti-TNF* | *No anti-TNF* |  |  |  |
|  | Sullivan et al | 2013 | 5.7 | 5.4 |  |  |  |
| Sleep Quality |  |  | *PSQI ≤5* | *PSQI ≥5* |  |  |  |
|  | Nie et al | 2018 | **2.95** | **4.95** |  |  | **<0.001** |
| Education |  |  | *<9 yrs* | *>9 yrs* |  |  |  |
|  | Li et al* | 2019 | 2.92 | 1.75 |  |  | N/S |
|  |  |  | *Low* | *Medium* | *High* |  |  |
|  | Bandinelli et al | 2016 | 10.28 | 8.578 | 7.253 |  | 0.0763 |
|  |  |  | *0-8 years* | *9-11* | *12-13* | *14-15* |  |
|  | **Dincer et al** | **2007** | **12** | **6.28** | **4.96** | **4.55** | **0.0018*** |
| Employment |  |  | *Full disability pension* | *No disability pension* |  |  |  |
|  | Forejtova et al* | 2012 | 7.5 | 7.5 |  |  | 0.021 |
|  |  |  | *Manual* | *Non-manual* |  |  |  |
|  | Bandinelli et al | 2016 | 10.54 | 8.275 |  |  | 0.0476 |
|  |  |  | *Work disability* | *No work disability* |  |  |  |
|  | **Abdul-Sattar et al** | **2014** | **4** | **8** |  |  | **<0.001** |
|  |  |  | *Work, no change* | *Work-disabled, change in job* | *Work-disabled- permanently disabled* |  |  |
|  | Cakar et al | 2009 | 3.7 | 7.3 | 7.8 |  | 0.028 |

*=Diagnostic delay reported as median
